# Research on the Effects of Entrepreneurial Education and Entrepreneurial Self-Efficacy on College Students’ Entrepreneurial Intention

**DOI:** 10.3389/fpsyg.2019.00869

**Published:** 2019-04-24

**Authors:** Xianyue Liu, Chunpei Lin, Guanxi Zhao, Dali Zhao

**Affiliations:** ^1^Business School, Huaqiao University, Quanzhou, China; ^2^College of Economics and Management, North China University of Technology, Beijing, China

**Keywords:** entrepreneurial education, entrepreneurial self-efficacy, entrepreneurial attitude, entrepreneurial intention, college students

## Abstract

Entrepreneurship is one of the important engines of economic development. Under the influence of policy encouragement and economic situation, college students have become the emerging entrepreneurial subjects. Studying the factors influencing their willingness to innovate is conducive to improving the entrepreneurial status and performance. From the perspective of planned behavior theory, this paper analyzes the effects of college students’ entrepreneurship education and self-efficacy on their entrepreneurial intention. Using a sample of 327 college students in China, we test the hypotheses, and get some results. Firstly, college students’ entrepreneurial education has a significant positive effect on their entrepreneurial intention, but has no obvious effect on the entrepreneurial attitude. Secondly, college students’ entrepreneurial self-efficacy has a significant positive effect on the entrepreneurial attitude and entrepreneurial intention, and the entrepreneurial attitude plays a partial intermediary role in the relationship between entrepreneurial self-efficacy and entrepreneurial intention.

## Introduction

Following the support of the Internet and mobile technologies, an increase in entrepreneurship has been observed among the college students in China. The trend is further intensified by encouragement from the government. A report indicated that, in China the proportion of college graduates engaged in entrepreneurship rose steadily, from 2.0% in 2012 to 3.0% in 2016, and 2.9 percent of college graduates started their own businesses 6 months later in 2017, with an increase of 1.9 percentage points over the past decade, compared with 1.0 percent in MyCOS (2018). Moreover, the government calls for ‘mass entrepreneurship and innovation’ and ‘optimizing the environment and providing uninterrupted services to increase the proportion of the college students’ employment and entrepreneurship. Under this context, the universities in China have begun to offer innovative entrepreneurship training courses and services in the form of entrepreneurship programs, competitions, and sandbox simulations to raise the entrepreneurial intention among college students and improve their success rates.

Entrepreneurial intention is the most important predictor for an individual’s entrepreneurial behavior (Lüthje and Franke, 2003). From various perspectives, numerous studies have examined the factors that influence entrepreneurial intention. Studies examining entrepreneurship characteristics have identified age, gender, level of education, personality traits, entrepreneurial knowledge and ability, desire for achievement, spirit of adventure, and value orientation as critical factors (Brockhaus, 1980; Krueger and Brazeal, 1994; Murry and MacMillan, 1998; Becherer and Maurer, 1999). Meanwhile the opposite of proactive personality also have important effect on entrepreneurial intention, such as narcissism, psychopathy and Machiavellianism (Wu et al., 2019). Also, from the perspective of entrepreneurial environment, studies have identified family education, market accessibility, entrepreneurial orientation from the government and related incentive policies and training services, and regional entrepreneurial atmosphere as fundamental factors (Bruno and Tyebjee, 1982; Gartner, 1985; Westhead and Batstone, 1999). Additionally, regarding the interactive internal and external factors, researchers have proposed many psychological models of entrepreneurship to explain the entrepreneurial intention and behaviors of an individual. Of these models, the Theory of Planned Behavior is the most influential (Bird, 1988; Ajzen, 1991). In the Theory of Planned Behavior, entrepreneurial intention is determined by an individual’s attitude and subjective norm attitude, subjective norms, and perceived behavioral control. Although entrepreneurial education provided by universities is a primary source of entrepreneurial knowledge and skills to improves an individual’s quality of entrepreneurship and a key factor in entrepreneurial success (Chusimir, 1988; Galloway and Brown, 2002), it has not received due attention in the aforementioned psychological models. Therefore, this paper applied the Theory of Planned Behavior to examine the effects of entrepreneurial education and self-efficacy on college students’ entrepreneurial intention.

## Theoretical Analysis and Research Hypotheses

### The Effect of Entrepreneurial Education on the College Students’ Entrepreneurial Attitude and Intention

Drucker (1985) stated that entrepreneurship can be learned through training. Kuratko (2014) also confirms that the personality traits, abilities, and skills required to become entrepreneurs can be acquired through training. In this paper, it was hypothesized that entrepreneurship can be learned from ‘learning by doing’ in the process of becoming an entrepreneur, and also from related entrepreneurship courses. Entrepreneurial education aims to develop and enhance the quality of entrepreneurship, ambition, drive, and pioneering and adventurous spirit for the college students to prepare for a certain career, enterprise, or business plan. It also aims to develop the strategic resources and abilities required by an entrepreneur and help him discover and recognize the business opportunities. In recent years, the universities and related external institutions have hosted various entrepreneurial training program, and these program have gradually gained attention. And the participants in such program are mostly the prospective entrepreneurs or the entrepreneurs who perceive that they lack the related knowledge and abilities after starting a business. These participants expect to improve their entrepreneurial capabilities through such programs, and acquire the ability to create, grasp, and pursue opportunities (Roomi and Harrison, 2008).

In social psychology, attitude is defined as the characterisation of personal cognition, including the subjective assessment of self, others, affairs, activities, events, and so on. And it has an important influence on an individual’s reactions and behavior. Entrepreneurial education is believed to inspire an individual’s entrepreneurism and further affect their perception of and passion for entrepreneurship (O’Cinneide and Garavan, 1994). Lundström and Stevenson (2005) also argued that entrepreneurial education and training can affect people’s attitudes and behavioral intentions toward entrepreneurship, and improve their management abilities. In summary, the purpose of entrepreneurial education is to help people to develop entrepreneurial capability, which is a combination of knowledge, attitude, and numerous abilities (Fiet, 2001). Therefore, this paper suggests that the attitude toward entrepreneurship is closely related to an individual’s entrepreneurial experience, and the self-learning of and external training in entrepreneurship can strengthen college students’ cognition of the entrepreneurial process, and imbue them with a proactive attitude.

The entrepreneurs consider a strong entrepreneurial intention as a prerequisite in starting a new business. Entrepreneurial intention is an individual’s conviction to make preparations for a new business and actually follow through on this goal (Krueger et al., 2000). It can be regarded as the planned behavior for starting a new business, which is a prerequisite for potential entrepreneurs (Wu and Jung, 2008; Premalatha, 2010). McMullan et al. (2002) found that entrepreneurial education can stimulate the ideas and behavior necessary for entrepreneurship. Botha (2006) suggested that the goal of entrepreneurial education should be to foster the individuals’ entrepreneurial intention. Through empirical research, Katz (2007) revealed that entrepreneurial education and training can strengthen an individual’s entrepreneurial intention. Through a similar research approach, Barbosa et al. (2008) found that entrepreneurial training can strengthen the entrepreneurs’ entrepreneurial intention and behavior and improve their entrepreneurial performance. Therefore, we believe that for college students with entrepreneurial intention or potential, entrepreneurial education can help them develop entrepreneurial knowledge and skills, and improve their success rate of starting a business.

Based on the above analysis, this paper proposes the following hypotheses:

Hypothesis 1: Entrepreneurial education for college students has a positive effect on their entrepreneurial attitude.
Hypothesis 2: Entrepreneurial education for college students has a positive effect on their entrepreneurial intention.

### The Effect of the College Students’ Entrepreneurial Self-Efficacy on Their Entrepreneurial Attitude and Intention

Bandura (1986) defined self-efficacy as the self-judgment on one’s ability to execute a series of actions to achieve a desired goal. In other words, it does not emphasize the skills an individual possesses, but rather an individual’s self-assessment on the ability to use these skills to achieve a goal. Later, Benight and Bandura (2004) further proposed that an individual’s self-efficacy can regulate the action through his or her own cognitive, motivational, affective, and decisional processes. As such, an individual’s self-perception self-efficacy of the ability to complete a task has a significant effect on their actual ability to play (Bandura, 1978). Drawing on these studies, we define entrepreneurial self-efficacy as an entrepreneur’s self-confidence regarding their ability to start business and his or her belief in possessing the abilities required to do so, that is, the entrepreneur’s self-confidence that he should be able to complete a certain task related to entrepreneurship.

According to the Expectancy-Value Theory, attitude is positively correlated with the product of behavioral beliefs and outcome evaluation. As stated by Ajzen (1991), when individuals believe that performing a certain action can achieve a positive result, they would exhibit a stronger preference toward this action, and then develop the intention to perform it. As we know, individuals’ self-efficacy can affect their goal-setting behavior and their conviction to achieve this goal. For the entrepreneurs, their self-efficacy has a high correlation with their perceived self-ability and entrepreneurial actions (Boyd and Vozikis, 1994). Thus, in this study, it is suggested that when entrepreneurs believe they have the ability to perform and achieve an entrepreneurial task, they will be more resolute in their entrepreneurial attitude.

An effective behavior not only requires appropriate knowledge, skills, and good attitude, but also need a conviction in the ability to making full use of them (Bandura, 1999). Krueger and Brazeal (1994) believe that self-efficacy is a predictor of entrepreneurial achievement, and entrepreneurial intention is, to some extent, determined by the attraction to entrepreneurship and individuals’ entrepreneurial self-efficacy. In other words, self-efficacy is the key factor that can help the entrepreneurs to overcome difficulties and face challenges in the process of entrepreneurship, and has a significant influence on their entrepreneurial intention. Furthermore, the existing studies have also revealed that entrepreneurial self-efficacy has a positive effect on entrepreneurial intention (Kolvereid, 1996; Miller et al., 2012; Martin et al., 2013). Therefore, in this study, we believe that, for the college students, self-efficacy is also positively correlated with entrepreneurial intention; that is, their confidence in having the requisite resources and abilities for starting a new business has a positive effect on the intention to achieve this goal. Hence, the college students who have a higher level of self-efficacy will also have a stronger entrepreneurial intention.

Based on the above analysis, this paper proposes the following hypotheses:

Hypothesis 3: College students’ entrepreneurial self-efficacy has a positive effect on their entrepreneurial attitude.
Hypothesis 4: College students’ entrepreneurial self-efficacy has a positive effect on their entrepreneurial intention.

Mitchell et al. (2003) considered entrepreneurial attitude to be the entrepreneurs’ opinions about the adaptability, abilities, and actions in the entrepreneurial process. Wyk et al. (2003) defined entrepreneurial attitude as changeable but predictable entrepreneurial thoughts and emotions. According to the Theory of Planned Behavior proposed by Ajzen and Fishbein (1980), individuals’ attitude affects their ultimate behavioral intention. Additionally, individuals’ behavioral attitude is a predictor of their behavioral intention (Petty and Briñol, 2010), as behavioral attitude can explain more than 50% of the variances in behavioral intentions (Prodan and Drnovsek, 2010). Koh’s (1995) empirical study on 200 business college students in Hong Kong demonstrated that entrepreneurial intention is significantly associated with the need for achievement, propensity to take risks, tolerance of ambiguity, and innovativeness. From a combined analysis of these studies, we propose that entrepreneurial attitude is formed from an individual’s subjective cognition of entrepreneurship and their emotions, and it has an important effect on an individual’s behavioral intention.

In the Theory of Reasoned Action and Theory of Planned Behavior, behavioral attitude is regarded as a key variable that explains behavioral intention. Both theories suggest that the basic belief in behavior and the evaluation of the possible consequences of performing that behavior are the antecedents of behavioral attitude (Ajzen and Fishbein, 1980; Ajzen, 1991). Entrepreneurial self-efficacy reflects an individual’s confidence in and judgment of the resources, abilities, and desired results involved in an entrepreneurial process (Bandura, 1986; Benight and Bandura, 2004). Additionally, Davidson (1995) considered entrepreneurial attitude as a mediator between personal background and entrepreneurial beliefs to investigate how they affect entrepreneurial intention and personal background included the persons’ educational status and related entrepreneurial experiences. While the entrepreneurial education in this paper, which encompasses both college students’ entrepreneurial self-learning and the entrepreneurial courses and training provided by universities or relevant educational institutions, can provide these students with Entrepreneurship education, training, knowledge and ability, and can reflect an individual’s entrepreneurial learning behavior more comprehensively. That is, the entrepreneurial education actually reflects the individuals’ entrepreneurship background. Therefore, in this paper, college students’ entrepreneurial attitude is considered to mediate the influence of both of entrepreneurial education and entrepreneurial self-efficacy on entrepreneurial intention.

Based on the above analysis, this paper proposes the following hypotheses:

Hypothesis 5: College students’ entrepreneurial attitude mediates the relationship between entrepreneurial education and entrepreneurial intention.
Hypothesis 6: College students’ entrepreneurial attitude mediates the relationship between entrepreneurial self-efficacy and entrepreneurial intention.

## Research Design

### Measurement of Variables

Existing studies mostly measure entrepreneurial education from individual’s level of education and entrepreneurial experiences and knowledge (Davidson, 1995). In this study, entrepreneurial education not only encompassed the entrepreneurial courses and practical training provided by external entities such as schools, but also college students’ entrepreneurial self-learning. These are measured by four question items, such as ‘I invest much time and energy in studying the latest developments in business management’ and ‘I have received some entrepreneurial education or training,’ and so on. Entrepreneurial self-efficacy is measured by four question items adopted from the scales in the studies of Cooper and Lucas (2006) and Barakat et al. (2014) such as ‘I am able to choose suitable employees for my own business’ and ‘I am able to apply innovative ideas to inspire entrepreneurial partners’ initiative, and so on. Entrepreneurial attitude is measured by five question items adopted from the study of Robinson et al. (1991) such as ‘I am strongly motivated to achieve career success’ and ‘I keep looking for new methods that can improve my performance,’ and so on. Entrepreneurial intention is measured by four question items adopted from the study of Thompson (2009), such as ‘I think I will start my own business in the future’ and ‘If given the chance to make a free decision, I will choose to start my own business, and so on. All of these items are modified to suit Chinese cultural context, and the measurement is based on a 5-point Likert scale, with 1 point denoting ‘strongly disagree’ and 5 points denoting “strongly agree.”

In addition, Brockhaus (1980) suggested that age, gender, and level of education could affect an individual’s entrepreneurial intention. And the grade year of college students can to some extent reflect their level of education. Thus, the age, gender, and grade of college students were used in this paper as control variables. The reported age of the responders is introduced into the model in the form of a natural logarithm; their gender is converted into a dummy variable, with 1 denoting male and 0 denoting female; and their grade is converted by using a 7-point scale, with 1 representing a freshman and 7 representing a 3rd-year graduate student.

### Sample Distribution

The data used in this study are collected through a questionnaire survey. More than 800 copies of the questionnaire were distributed among the students of universities in the Fujian Province, and a total of 412 copies are returned, posting a recovery rate of approximately 51.5%. After excluding 85 invalid copies that omitted answer more than five question items or where students gave the same answer to at least 10 consecutive question items, we get 327 valid copies, posting an effective response rate of 79.3%. The sample properties are shown in Table 1.

**Table 1:** Statistics of features of students sample.

Gender		Age		Grade		Major	
Male	66.3%	<21	7.9%	1	1.5%	Arts and Humanities	81%
female	36.7%	21	16.8%	2	7.3%	Science and Engineering	19%
		22	37.0%	3	27.2%		
		23	26.0%	4	63.3%		
				5	0.3%		
				6	0		
				7	0.3%		


## Empirical Results and Analyses

### Reliability and Validity Analysis

We test the reliability by using internal consistency reliability and composite reliability. Table 2 shows that all the Cronbach’s alpha are greater than 0.70, and all the value of composite reliability are all exceeds the 0.60 benchmark.

**Table 2:** Results of reliability and validity analysis.

Variables	Items	Factor	CITC	Cronbach’s	Cronbach’s	CR	AVE
		loading		α if item	α		
				deleted			
Entrepreneurial education	I invest much time and energy in studying the latest developments in business management.	0.648	0.595	0.851	0.853	0.856	0.601
	I have received some entrepreneurial education or training.	0.839	0.749	0.788			
	I have a lot of knowledge about management (entrepreneurship).	0.838	0.756	0.788			
	I have many entrepreneurial experiences.	0.760	0.690	0.815			
Entrepreneurial self-efficacy	I am able to choose suitable employees for my own business.	0.734	0.656	0.839	0.858	0.859	0.605
	I am able to apply innovative ideas to inspire entrepreneurial partners.	0.810	0.736	0.806			
	I can write a clear and complete business plan.	0.770	0.697	0.822			
	I can make a clear plan for the future development direction of entrepreneurship.	0.795	0.726	0.810			
Entrepreneurial attitude	I am strongly motivated to achieve career success.	0.717	0.659	0.826	0.850	0.854	0.540
	The pursuit of innovation is my style of doing things.	0.758	0.685	0.813			
	I believe that as long as I work hard, things will be successful.	0.720	0.666	0.819			
	I can do anything well.	0.731	0.661	0.822			
	I keep looking for new methods that can improve my performance.	0.748	0.664	0.819			
Entrepreneurial intention	I think I will start my own business in the future.	0.863	0.794	0.829	0.881	0.883	0.656
	I’ve considered running my own company.	0.823	0.752	0.844			
	If given the chance to make a free decision, I will choose to start my own business.	0.824	0.760	0.841			
	Considering the current situation and various restrictions (such as capital), I will still choose to start my own business.	0.724	0.668	0.876			


The majority of items used in the present study are adopted from tested and proven scales, and are modified to suit the Chinese cultural context, so the scale exhibites good content validity. Then exploratory factor analysis (EFA) conducted by AMOS 22.0 is used to test the construct validity of the questionnaire. The Kaiser-Meyer-Olkin value of the 17 items is 0.908, and the chi-square value in the Bartlett’s test of sphericity is 3131.839 (degree of freedom = 136), which showed statistical significance. All of these indicate that there are common factors among the correlation matrices, and it’s suitable for factor analysis. The factors are identified by using the methods of principal component analysis and varimax rotation, and the number of factors is determined by the Eigenvalue greater than one. These analyses provided four factors: entrepreneurial attitude, entrepreneurial intention, entrepreneurial self-efficacy, and entrepreneurial education. Their cumulative explained variance is 70.159%, and the factor loadings after rotation all exceed 0.563. Therefore, the scale is of good construct validity. Additionally, a confirmatory factor analysis is used to test the discriminant validity of the four-factor model. Compared with the one-factor and three-factor models, the four-factor model is the most ideal for the fitting with actual data (χ^2^/df = 2.75; CFI = 0.935; GFI = 0.898; TLI = 0.922; IFI = 0.936; RMSEA = 0.073). It indicates that the four factors exhibited favorable discriminant validity and they genuinely represented four different constructs. Therefore, it’s suitable to proceed to the next step: correlation and regression analyses.

Table 3 presents the mean and standard deviation of the variables and the Pearson’s correlation coefficient between the variables. These correlation analysis results reveal that the correlation coefficients among entrepreneurial education, entrepreneurial self-efficacy, and entrepreneurial attitude and entrepreneurial intention are all statistically significant at different statistical levels. The average variances extracted (AVE) are greater than 0.50, and exceed the absolute value of other variables’ correlation coefficients, so all the above measures demonstrate adequate validity and reliability.

**Table 3:** Descriptive statistics and Pearson’s relation among variables.

Variable	Mean	SD	1	2	3	4	5	6	7
(1) Gender	1.37	0.483	–						
(2) Age	22.19	1.215	008	–					
(3) Grade	3.55	0.729	099	0.300**	–				
(4) Entrepreneurial education	2.652	0.968	-0.125*	0.093	0.065	0.775			
(5) Entrepreneurial self-efficacy	3.376	0.799	0.006	0.066	0.128*	0.512**	0.778		
(6) Entrepreneurial attitude	3.557	0.822	-0.046	-0.051	0.151**	0.271**	0.554**	0.735	
(7) Entrepreneurial intention	3.055	0.986	-0.100	0.003	0.043	0.490**	0.615**	0.445**	0.810


*^∗^Correlation is significant at the 0.05 level (2-tailed); ^∗∗^Correlation is significant at the 0.01 level (2-tailed); numbers on the diagonal are the square roots of AVE.*

### Common Method Bias Test

Although mature scales were selected in this study, the problem of data source identity could not be completely avoided, which may lead to the emergence of common method deviation. So common method bias needs to be further verified. By using Harman single factor test, the results showed that all the items are aggregated into four factors without rotation, each factor has an Eigenvalue greater than one, and the cumulative variance contribution rate is 70.159%, the first Eigenvalue is 7.246, variance contribution rate is 42.623%, the proportion is less than 50% of the total explanatory variables. Also, the results of collinearity test show that, 1.039 ≤ VIF ≤ 1.840, 0.544 ≤ TOL ≤ 0.962. Therefore, this study is not greatly affected by the common method bias and collinearity.

### Regression Analysis and Discussion

According to hierarchical regression procedure, we first set gender, age, and grade as control variables, and then set entrepreneurial education and entrepreneurial self-efficacy as independent variables, and entrepreneurial attitude and entrepreneurial intention as dependent variables. After that, we carried out the regression analysis. And the results are presented in Table 4.

**Table 4:** Regression analysis on the effects of entrepreneurial education and entrepreneurial self-efficacy on entrepreneurial attitude and entrepreneurial intention.

Variable	Entrepreneurial attitude		Entrepreneurial intention		
	Model 1	Model 2	Model 3	Model 4	Model 5
Gender	0.065	0.064	0.105	0.072+	0.063
Age	-0.106+	-0.121*	-0.017	-0.050	-0.031
Grade	0.191**	0.125*	0.058	-0.014	-0.033
Entrepreneurial education		-0.018		0.229***	0.231***
Entrepreneurial self-efficacy		0.556***		0.504***	0.420***
Entrepreneurial attitude					0.150**
R^2^	0.037	0.331	0.013	0.428	0.443
Adjusted R^2^	0.028	0.320	0.004	0.420	0.433
F	4.132	31.712	1.414	48.117	42.491


*Model 2 vs. Model 1; Model 4 vs. Model 3; Model 5 vs. Model 4. ^∗∗∗^denotes p < 0.001; ^∗∗^denotes p < 0.01; ^∗^denotes p < 0.05; and ^+^denotes p < 0.1.*

Models 1 and 2 are used to examine the direct effect of entrepreneurial education and entrepreneurial self-efficacy on college students’ entrepreneurial attitude. Model 1 investigate the effects of the three control variables of gender, age, and grade on entrepreneurial attitude, whereas Model 2 analyzed the effects of the control variables and the two independent variables of entrepreneurial education and entrepreneurial self-efficacy. Apparently, based on the control variables, entrepreneurial education and entrepreneurial attitude do not exhibit a significant correlation (β = -0.018, *p* > 0.7). Therefore, Hypothesis 1 is not supported. A possible reason could be that the college students still require a psychological transformation mechanism to internalize the entrepreneurial knowledge, skills, and experiences acquired from entrepreneurial education into entrepreneurial attitude. And this may be related to entrepreneurial self-efficacy. This is because, as an individual’s perspective and preference over entrepreneurship, entrepreneurial attitude is largely decided by the individual’s affections and cognition of entrepreneurship. Therefore, the impact of entrepreneurial education on entrepreneurial attitude may need to be demonstrated through the impact of entrepreneurial self-efficacy. Additionally, Davidson (1995) also pointed out that an individual’s attitude toward ‘playing the game of entrepreneurship” is a spontaneous motivation arising from an individual’s psychological orientation, i.e., entrepreneurship traits. Thus, proactive personality can be included in the model to explore the effect of entrepreneurial education on entrepreneurial attitude and intention. The results also demonstrated that college students with higher entrepreneurial self-efficacy exhibited greater entrepreneurial attitude (β = 0.556, *p* < 0.001). This supported Hypothesis 3, and the views of Ajzen (1991); Boyd and Vozikis (1994), that is, when college students are fully confident in their entrepreneurial resources and abilities, they are more resolute in their entrepreneurial attitude.

Models 3 and 4 are used to examine the direct effect of entrepreneurial education and self-efficacy on college students’ entrepreneurial intention. Model 3 investigate the effects of the three control variables of gender, age, and grade on entrepreneurial intention, whereas Model 4 examined the effects of the control variables and the two independent variables of entrepreneurial education and entrepreneurial self-efficacy on entrepreneurial intention. The results show that, based on the control variables, the more intensive the entrepreneurial education was, the stronger the entrepreneurial intention would be (β = 0.229, *p* < 0.001). This supported Hypothesis 2, and achieved empirical results similar to those of McMullan et al. (2002); Botha (2006), and Katz (2007). Thus, extensive entrepreneurial education can greatly stimulate college students’ entrepreneurial intention. Similarly, when college students exhibit high entrepreneurial self-efficacy, they also have strong entrepreneurial intentions (β = 0.504, *p* < 0.001), which supported Hypothesis 4. This suggests that adequate entrepreneurial self-efficacy can strengthen college students’ confidence in their entrepreneurial abilities, and further inspire their conviction and intention in entrepreneurship. These results support the view of Krueger and Brazeal (1994), and are consistent with the findings of Kolvereid (1996); Miller et al. (2012), and Martin et al. (2013).

Finally, the mediating effect of entrepreneurial attitude is tested according to the procedure proposed by Baron and Kenny (1986). First, the significant correlation between the independent and dependent variables is confirmed, followed by the significant correlation between independent variables and the mediator. As is known from the preceding analysis, based on the control variables, the significant correlation between entrepreneurial education and college students’ attitude is not confirmed, which don’t satisfied the prerequisite for mediation analysis, and Hypothesis 5 is not be proved; while college students’ entrepreneurial self-efficacy is positively correlated with entrepreneurial attitude and intention, ensuring their feasibility for mediation analysis. Further, entrepreneurial attitude is introduced on the basis of Model 4 to examine its mediating effect, and the results are provided in Model 5 in Table 3. Compared with Model 4, the regression coefficient of entrepreneurial self-efficacy has an obvious decline (β = 0.504 → 0.420) and reached statistical significance. This indicated a partial mediating effect, and Hypothesis 6 is supported. This result demonstrates that college students’ entrepreneurial self-efficacy not only has a direct and significant positive effect on entrepreneurial intention, but also exercises an indirect positive effect through entrepreneurial attitude.

## Discussion and Conclusion

This study revealed that entrepreneurial education has a significant and positive effect on college students’ entrepreneurial intention, but does not have a significant effect on entrepreneurial attitude; entrepreneurial self-efficacy has a significant and positive effect on both entrepreneurial attitude and entrepreneurial intention, and entrepreneurial attitude further partly mediates the relationships between entrepreneurial self-efficacy and entrepreneurial intention. These findings contribute to the development of entrepreneurship theories, and provide important inspiration for Chinese college students’ entrepreneurship and the practice of entrepreneurship education in universities and related training institutions.

The findings indicate theoretical significance. First, entrepreneurial education and entrepreneurial self-efficacy play critical roles in stimulating college students’ entrepreneurial intention, but the psychological model used in this study did not enable an adequate exploration of their relationships. Previous models of entrepreneurial psychology mainly focused on the influence of individual’s inherent characteristics, education level, family business experience, entrepreneurial career expectation prediction and other aspects on the entrepreneurial intention. This study uses entrepreneurial education and self-efficacy as independent variables, and entrepreneurial attitude as a mediator, to construct an influencing mechanism model to determine college students’ entrepreneurial intention. It further tested and supplemented the Theory of Planned Behavior from the perspective of learning and self-efficacy. The findings provide empirical evidence to support existing theories, and will also serve as a valuable reference for follow-up studies.

Concerning management practices, these findings suggest that entrepreneurial education and self-efficacy can effectively inspire college students’ entrepreneurial intention. Entrepreneurship is an activity that requires management, and through the provision of entrepreneurial education in the form of self-learning and taught courses, college students are able to acquire the knowledge, skills, and practical experience required for the entrepreneurial process which can then improve their entrepreneurial intention. The stronger the perceived entrepreneurial self-efficacy, the more effective will college students exert their innate entrepreneurial abilities, strengthen their entrepreneurial potential, and inspire their entrepreneurial confidence and passion. These conclusions suggest that universities and other relevant educational institutions should pay more attention to the combination of self-learning and external training in entrepreneurship, as well as the perception of entrepreneurial self-efficacy, so as to enrich the connotation of entrepreneurship education and improve its effectiveness.

## Limitations and Future Research

This study has some limitations. First, sample selection of this study used convenience sampling instead of random sampling, and all the students are come from single province, which may affect the representativeness and universality of the results. Future research might expand sample or compare samples from cross-region in the same country or different countries, which have different culture, social norms and other socioeconomic contexts (Zampetakis et al., 2015; Gorgievski et al., 2017; Dukhon et al., 2018). These contextual factors might have significant influence on special sample’s entrepreneurial intention. For example, Arshad et al. (2019) studied the influence of collectivist orientation in students’ entrepreneurial intention with Pakistani sample.

Second, this study did not consider the effect of educational background on entrepreneurial attitude and intention. Existing studies have found that major and entrepreneurship courses are important contextual factors in educational system, which might influence students’ thinking about their future careers and career options, and result in different attitude and intention (Misoska et al., 2016). Meanwhile some comparative analysis showed that subjective norms have different influence on entrepreneurial intention in separate categories of students, who have different educational background (Maresch et al., 2016). Sample of this study did not classify the educational background detailed, which maybe the reason why Hypothesis 1 was not supported in this study. Therefore, future research can analyze the influence mechanism of entrepreneurial education on attitude and intention in different educational background, which might helpful to entrepreneurial educational system in curriculum design, and policy provision.

Third, entrepreneurial education is the combination of entrepreneurship and pedagogy, therefore, which have been measured by extant research. In this study, the measurements of entrepreneurial education use four items, including courses, practical training provided by schools and self-learning. In future, dividing these items into two variables according to the Theory of Planned Behavior, and also introducing personality traits variables that can affect entrepreneurial attitude and intention (e.g., proactive personality), might be worthy study.

## Ethics Statement

This research was carried out in accordance with the recommendations of moral rule for empirical research, and approved by the Academic Committee of Business School of Huaqiao University; meanwhile all the survey respondents were given written informed consent.

## Author Contributions

All authors listed have made a substantial, direct and intellectual contribution to the work, and approved it for publication.

## Conflict of Interest Statement

The authors declare that the research was conducted in the absence of any commercial or financial relationships that could be construed as a potential conflict of interest.
